# The Effectiveness of Online Mental Health First Aid Training in Community Rugby: A Mixed-Methods Approach

**DOI:** 10.3390/ijerph20075391

**Published:** 2023-04-04

**Authors:** Suzanna Russell, Vincent Kelly, Remco Polman, Matthew Warren-James

**Affiliations:** 1 Sports Performance, Recovery, Injury and New Technologies (SPRINT) Research Centre, School of Behavioural and Health Sciences, Australian Catholic University, Brisbane, QLD 4014, Australia; 2 School of Exercise and Nutrition Sciences, Queensland University of Technology, Brisbane, QLD 4006, Australia; 3 Institute of Health and Wellbeing, Federation University, Melbourne, VIC 3806, Australia; 4 School of Clinical Sciences, Queensland University of Technology, Brisbane, QLD 4006, Australia; 5 School of Nursing, Midwifery and Paramedicine, University of the Sunshine Coast, Sunshine Coast, QLD 4556, Australia

**Keywords:** mental health literacy, mental health training, mental health awareness, early identification, Mental Health First Aid, sport, rugby, community, stigma, symptoms

## Abstract

Mental Health First Aid (MHFA) training exists to improve supportive behaviours towards peers, increase mental health literacy, and reduce stigma. Community sport clubs have potential to successfully deliver mental health programs. This study investigated the effectiveness of online MHFA training undertaken by members of the rugby community and evaluated the feasibility and usefulness of the online delivery mode and users’ engagement with it. A mixed-methods approach was used to provide depth of understanding through qualitative analysis, combined with quantitative outcomes. Online surveys examining participants’ knowledge and perceptions were administered pre- and post-MHFA training. Significant improvements (*p* < 0.05) across all assessed domains were observed post- compared to pre-MHFA training. A large effect size was identified in relation to advice giving and sign and symptom identification. A moderate effect size pre- to post-improvement was identified for users’ perceptions of therapy’s effectiveness, the ability of people with severe mental health conditions to recover, and benefit of a healthcare professional. Participants endorsed the MHFA program to improve mental health literacy, advance non-technical skills, and improve confidence. MHFA training can increase the awareness and knowledge of mental health issues in key individuals in community sport clubs and enable them to aid people with mental health concerns. Online MHFA training is associated with improved mental health literacy and may be a suitable and economically sustainable model for community sport.

## 1. Introduction

Approximately one in two Australians will experience mental illness in their lifetime, and one in five will experience mental illness in any one year [1]. Suicide is the leading cause of death in those 15–44 years of age, with the highest rates occurring in young people aged 18–24 [2]. Further, mental and substance use disorders are the fourth leading cause of total disease burden in Australia [2]. Young people are at high risk of experiencing adverse mental health, with 50% of cases occurring before 14 years of age, and almost 75% occurring before 25 years of age. The most recent survey results show that 20% of Australians aged 16–34 years experience high or very high levels of psychological distress, more than twice the rate of those aged 65–85 years. Routine clinical evaluation data and self-reported mental health symptoms also demonstrate youth athlete populations to be at risk of mental health disorders or associated symptoms [3]. Young individuals are reluctant to seek professional mental healthcare [4]. Stigma, a lack of mental health literacy, and prior negative experiences of help seeking have been identified as important perceived barriers to help seeking in young athletes [5]. It is known that young individuals are more inclined to seek help if they have established and trusted relationships with potential help providers [4]. Youth athletes also report an established relationship and positive attitudes of others (e.g., coaches) as facilitators to help seeking [5].

Community sport clubs have been identified as organisations with large potential to effectively deliver mental health promotion and in turn prevent the development of mental health disorders [6]. In particular, adolescent males perceived sport and team sport to be engaging vehicles for supporting mental health [6]. The already established social networks in sporting clubs provide an ideal environment in which to deliver mental health awareness programs [7]. Despite this, considering its potential for the promotion of key mental health messages and delivery of education for best-practice management for mental illness identification and referral, sport is largely an underutilised vehicle for mental health communication, with few grassroots community-level sporting organisations actively engaged in mental health initiatives [8]. As young people prefer to seek help from someone they know and trust, individuals within the sporting community have the ability to improve early identification and advocate for appropriate referral to promote help seeking. 

Whilst coaches, managers, officials, other support staff and volunteers are in a position to positively impact the mental health of young people [9], they report limited mental health literacy and a lack of confidence in leading conversations [10]. By increasing the knowledge of mental health disorders and treatments, community sport staff may increase their confidence in effectively engaging with and appropriately managing youth mental health issues. Accordingly, a case for improvement in mental health education and practice in sporting communities exists. One such community-based sport, with a large participation base of over 117,000 registered male and female club players, is rugby union (Rugby Australia). With the potential to reach a large population of young people, community rugby clubs are positioned as suitable organisations wherein improvements in mental health literacy, confidence in early identification of symptoms and skills to effectively initiate and lead conversations around mental health may be beneficial to this end. 

Mental Health First Aid (MHFA) is a standardised, psychoeducational program developed to improve the mental health literacy of participants and empower them to approach and assist individuals who may be experiencing mental illness [11]. Improving mental health literacy can reduce stigma, a major barrier to help seeking [12]. Two separate meta-analyses synthesising 15 and 18 published evaluations of the MHFA program, respectively, suggest participation in MHFA training increases participants’ mental health knowledge (Glass’s Δ = 0.56 (95% CI = 0.38–0.74; *p* < 0.001)), attitudes (Glass’s Δ = 0.28 (95% CI = 0.22–0.35; *p* < 0.001)) and the helping behaviours of participants (Glass’s Δ = 0.25 (95% CI = 0.12–0.38; *p* < 0.001) [11,13]. The long-term benefits of MHFA training include improved mental health first aid knowledge, recognition of disorders and beliefs about effective treatments six months post initial training [11]. Furthermore, MHFA training has been shown to be beneficial in community sport, as evidenced by a 50% increased capacity to recognise mental illness and a 66% increase in confidence in responding to mental health difficulties in club leaders of Australian Rules Football [14]. MHFA training has also been shown to be effective in numerous communities with a high presence of young people, including members of the public [15,16], high-school teachers [17] and tertiary staff [18]. However, to the authors’ knowledge, beyond the aforementioned studies, to date no research has evaluated the impact or efficacy of MHFA training in the rugby community.

Standard MHFA training courses, such as those used in previously published research, are led by qualified MHFA trainers and delivered face-to-face across a duration of two days. In June 2020, MHFA Australia created an online MHFA community course, replacing the two-day face-to-face course, to meet the physical distancing requirements implemented as a result of the global coronavirus pandemic. This course required participants to undertake 5 to 7 h of online pre-learning followed by two 2.5 h MHFA instructor-led online sessions. Currently, there is no research that explores the efficacy of the new online standard MHFA course; however, one recent study has reported on the benefits of an online delivery mode over e-learning involving other MHFA courses [19]. Furthermore, to date, the impact and efficacy of the new online standard MHFA course has not been explored in relation to community rugby, or within any other community sporting context. 

The primary purpose of this study was to investigate the effectiveness of online MHFA training in enhancing participants’ knowledge and understanding around mental health, increasing their perceived ability to manage mental health crises, refer individuals suffering from mental illness to appropriate support systems, and reduce the stigma around mental health in the rugby community. Secondly, the feasibility, usefulness, and engagement of the online delivery mode as a method for MHFA training in community sporting organisations were evaluated.

## 2. Materials and Methods

A mixed-methods approach was used to provide depth of understanding through qualitative analysis, combined with quantitative outcomes, within the context of the work, which aimed to influence applied practice [20,21]. The data collection process included an online survey both pre- and post-MHFA training. Each online survey included Likert and open-response questions that examined participants’ knowledge and perceptions about the MHFA course (survey available upon reasonable request). The survey was provided via an electronic link (Qualtrics, Provo, Utah). Participants provided written informed consent prior to participation, including consenting to information obtained to be utilised for evaluation and research purposes. Participants were informed that all survey responses and MHFA instructors would be anonymous and non-identifiable to the members of the research team to reduce the potential of social influence response bias. Ethical approval was obtained from the Queensland University of Technology Human Research Ethics Committee (UHREC reference number: 2021000229), in accordance with the World Medical Association Declaration of Helsinki.

### 2.1. Participants

Participants who volunteered to engage in MHFA training were provided with the opportunity to partake in the study. Thus, a purposeful sampling approach was used, with all members of the Queensland Rugby Union community being provided with the opportunity to participate in MHFA training, the invitations for which were sent via email communications, promoted on posters, and spread by word-of-mouth. Inclusion criteria included any individuals over the age of 18 who identified as being a member of the Queensland Rugby community and chose to engage in MHFA training (n = 31). No exclusion criteria were applied. Participants ranged from 18 to 69 years (35.8 ± 13 years) and 42% self-identified as female, and 58% male. Participants self-identified as a range of ethnicities, including Australian (65.6%), European (21.9%), Polynesian (9.4%) and African (3.1%). The highest prior completed education level was reported and included no formal education (3.2%), high-school completion (9.7%), vocational qualification (9.7%), associate diploma (9.7%), undergraduate diploma (9.7), bachelor’s degree (35.5%), bachelor’s degree with honours (6.5%), postgraduate diploma (9.7%) and master’s degree (6.5%). Participants held various full-time (64.5%), part-time (12.9%) and casual (9.7%) roles within the rugby community. Positions included managers (38.7%), volunteers (19.4%), operational staff (12.9%), players (12.9%), coaches (9.7%) and officials (6.5%). A portion of the volunteers (12.9%) self-indicated themselves as unemployed. A priori power calculation (G*Power 3.1, Germany) [22] indicated a minimum sample size of 19 participants. The sample size obtained (n = 31) was further deemed appropriate to inform the feasibility, impact and efficacy of MHFA training as per previous research demonstrating the impact of MHFA using similar sample sizes in Australian rural football club leaders (n = 36) [14] and a farming community (n = 32) [23,24]. 

### 2.2. Survey Details

The pre-MHFA online survey consisted of 14 questions and was accessible one week prior to participation in the MHFA training course and closed prior to the commencement of training. It entailed demographic questions, including identification of previous mental health or alcohol and other drug use training and a series of mental health literacy and awareness questions to determine participants’ perceptions of their ability to know what advice to give, whether therapy is an effective treatment, the ability of people with severe mental health conditions to recover, the benefit that a healthcare professional can provide for someone with mental health problems, as well as their ability to identify signs and symptoms of someone struggling with their mental health. The scale was slightly modified from the psychometrically tested the Mental Health Knowledge Schedule (MAKS) instrument by omitting the paid employment item. The MAKS has previously been shown to be a brief and feasible instrument for assessing and tracking stigma-related mental health knowledge [25]. Immediately following the completion of MHFA training, participants were provided with the opportunity to complete the post-MHFA online survey which consisted of 28 questions. The adapted MAKS questions, as per the pre-MHFA online survey, were repeated, and an additional series of questions that aligned with the purpose and intent of MHFA training [26] were asked to evaluate MHFA training outcomes. These questions asked participants whether they felt MHFA training impacted; their knowledge of signs and symptoms, whether it had provided useful information about depression and anxiety, substance use, the provision of initial help for those struggling with mental health problems, the effectiveness of treatment for mental health problems, the provision of MHFA support in a crisis situation and how to refer others to seek support, and whether it was useful and engaging (i.e., was the program informing, engaging and relatable, and was the mode of delivery satisfactory?). The Likert response scales were scored on a scale of 1–5 (1 = strongly disagree, 2 = disagree, 3 = unsure, 4 = agree, 5 = strongly agree).

### 2.3. MHFA Training

The MHFA training program is an evidence-based curriculum, informed by guidelines developed using the Delphi consensus method. The MHFA training program used an online approach, which followed the standard MHFA Australia format of a self-paced e-learning component (5–7 h) and an instructor-led component using video conferencing via Zoom (two 2.5 h sessions) [27]. The e-learning component was delivered via a series of standard modules, completed asynchronously and presented in an interactive manner, including self-assessment tasks for participants to check their learning progress. The synchronous instructor-led component included interactive learning activities such as case-based learning, role play, and self-reflection. Following participation in the e-learning and instructor-led components of the program, participants undertook an online accreditation assessment where a pass resulted in each individual obtaining a 3-year MHFA accreditation. Participants were also provided with an MHFA course manual in a portable document or physical book format. Training was led by a qualified MHFA instructor (male, 38 years of age) with 3 years of experience teaching MHFA courses and 12 years of experience teaching health and paramedical sciences at the tertiary education level. Each course had a maximum of 12 participants to ensure quality of education and opportunity for engagement of participants. 

### 2.4. Analysis

#### 2.4.1. Statistical Analysis

Responses were analysed using R Foundation for Statistical Computing software (Version 1.4.1717, Vienna, Austria). Only responses where matched samples existed for both pre- and post-time points were included in the analyses (n = 31). Mean, standard deviation (SD) and interquartile range (IQR) values (first quartile, 25%; third quartile, 75%) were calculated for each pre–post variable: advice giving, therapy effectiveness, recovery from severe mental health problems, healthcare professional benefit, and signs and symptom identification. Mean and SD were also calculated for variables assessed at only the post-MHFA time point to quantify training outcomes (knowledge of signs and symptoms, useful information about depression and anxiety, useful information about substance use, knowledge about provision of initial help for those struggling with mental health problems, knowledge about effectiveness of treatment for mental health problems, knowledge about provision of MHFA support in a crisis situation, knowledge about how to refer others to seek support), and usefulness and engagement (Was the program informing, engaging and relatable and the mode of delivery satisfactory?) using the R package “DescTools” [23]. To identify potential differences in variables assessed at both pre- and post-MHFA training time points, a *t*-test for each variable was run using the function wilcox.test with Bonferroni correction for multiple comparisons. Alpha was set at 0.05. Cohen’s d effect size differences (ESs), assessed at both pre- and post-MHFA training time points, were also calculated using the R package “effect size” [28]. ESs were interpreted as 0.00 to 0.19 (trivial), 0.20 to 0.59 (small), 0.60 to 1.19 (moderate), 1.20 to 1.99 (large), 2.00 to 3.99 (very large) and ≥4.00 (extremely large) [29].

#### 2.4.2. Qualitative Analysis

The Braun and Clarke [30] six-phase process for thematic analysis was followed, which included familiarising oneself with the data, generating initial codes, searching for themes via reviewing, defining, and naming themes and producing the report and final thematic map. Participant quotes were used as evidence of the themes and subthemes, and to provide context for the reader. 

## 3. Results

### 3.1. Pre- and Post-MHFA Training Responses

**Figure 1:**
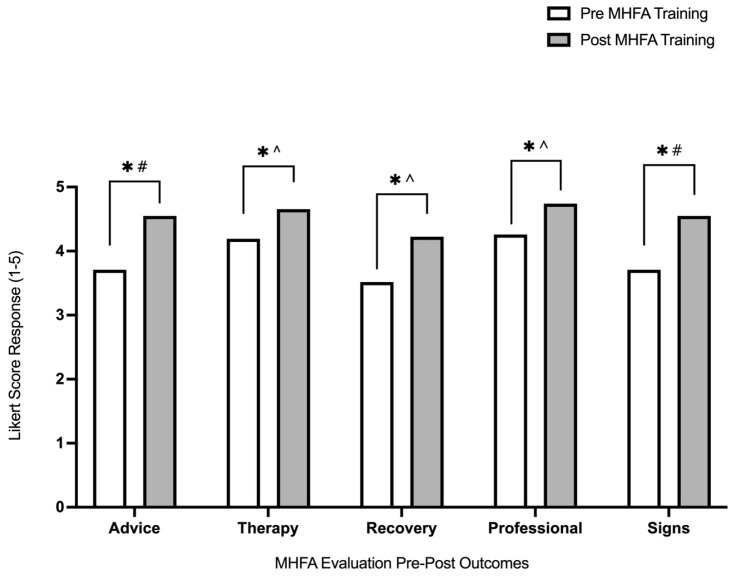
Pre- and post-MHFA training average Likert 1–5 score responses for advice, therapy, recovery, professional and signs and symptom categories. Likert scale response options: 1 = strongly disagree, 2 = disagree, 3 = unsure, 4 = agree, 5 = strongly agree. * = *p* < 0.01; # = large ES; ^ = moderate ES.

Pre–post MHFA training mean Likert scores are visualised in Figure 1. 

A significant improvement was found post-MHFA training compared to pre-training. For each domain, mean ± SD and IQR (first quartile–third quartile) pre–post scores are reported as follows: Advice Pre = 3.71 ± 0.74 (3.50–4.00), Post = 4.55 ± 0.57 (4.00–5.00) (*p* < 0.01); Therapy Pre = 4.19 ± 0.48 (4.00–4.00), Post = 4.66 ± 0.49 (4.00–5.00) (*p* < 0.01); Recovery Pre = 3.52 ± 0.81 (3.00–4.00), Post = 4.23 ± 0.80 (4.00–5.00) (*p* < 0.01); Professional Pre = 4.26 ± 0.58 (4.00–5.00), Post = 4.74 ± 0.44 (4.50–5.00) (*p* < 0.01); Signs Pre = 3.71 ± 0.64 (3.00–4.00), Post = 4.55 ± 0.51 (4.00–5.00) (*p* < 0.01). A large ES was found post-MHFA training compared to pre-training for knowing what advice to give (−1.27 [−1.82, −0.72]) and identification of signs and symptoms of someone struggling with their mental health (−1.45 [−2.01, −0.88]), and a moderate ES was found for perceptions of therapy being an effective treatment (−0.94 [−1.46, −0.41]), the ability of people with severe mental health conditions to recover (−0.88 [−1.40, −0.35]) and benefit that a healthcare professional can provide for someone with mental health problems (−0.94 [−1.46, −0.41]).

### 3.2. MHFA Outcome Evaluation 

Likert (1–5) evaluation scores of MHFA training outcomes demonstrate that post-MHFA training, participants either agreed (4) or strongly agreed (5) that the MHFA training provided useful information or equipped them with knowledge across several domains, as presented in Table 1. 

**Figure 2:**
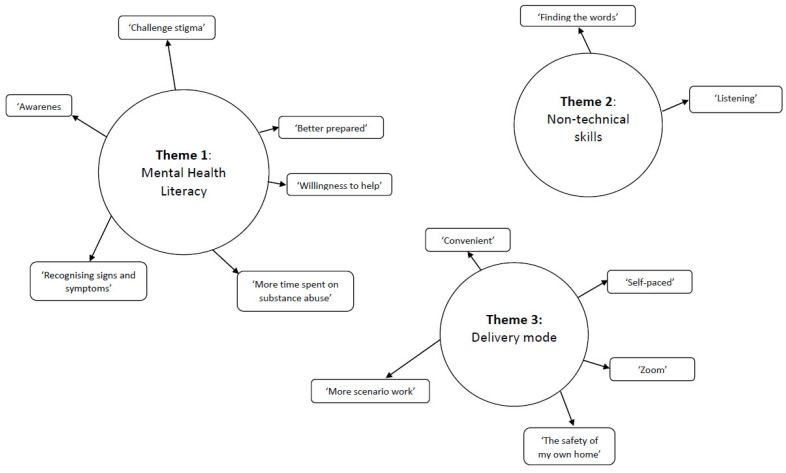
Final thematic map.

The final thematic map (Figure 2) provides a diagrammatic interpretation of the data and captures the essence of each of the three themes—mental health literacy, non-technical skills, and delivery mode—and their multiple associated subthemes. 

### 3.3. Mental Health Literacy 

Mean Likert (1–5) evaluation scores of MHFA training indicated participants found the MHFA training informative (4.67 ± 0.66), engaging (4.80 ± 0.41) and relatable (4.83 ± 0.38), and were satisfied with the online mode of delivery (4.60 ± 0.56).

Interpretation of open responses indicates the MHFA training provided useful information that enhanced participants’ mental health literacy through an awareness of the sequelae of mental illness:

“I gained more. I thought it would be another ‘flick through’ online course but it was very interactive, and I now feel like I have the tools to open up these conversations.”

“recognising signs and symptoms and knowing what hope and resources I can give them.”

Participants also highlighted that group discussion within MHFA training provided an opportunity to challenge the existing stigma around mental illness in club rugby:

“I think it will be great for the rugby community to be able to identify when someone might [be] struggling but also goes a long way to challenging the stigma around mental health.”

“……it allows people to reflect on how we can implement MHFA as a coach, …… and break down some of the barriers and views that are held.”

Improvements in mental health literacy were also perceived to enhance participants’ willingness to communicate with club members:

“Awareness—that in itself can help exponentially. Sharing information with parents in the club.”

Although most participants were happy with the content of the MHFA training, some participants indicated they would like more information about substance abuse given it is perceived to be prevalent in the rugby community:

“For the context of rugby in high performance maybe substance abuse and also the fact that the supplement industry isn’t regulated and may have banned substances.”

“More time spent on substance abuse.”

### 3.4. Non-Technical Skills

Another perceived benefit of engaging in MHFA training was the development of non-technical skills that may be useful when helping a person with a mental health challenge:

“gaining a better understanding of the framework for understanding and listening to people.”

“listen—ask open questions, leave space.”

Overall, after completing the MHFA training, participants reported feeling better prepared to help members of their own community, and more confident in their ability to help someone struggling with mental illness:

“better prepared to have the conversation.”

“to be brave enough to say something.”

“More people being equipped with the knowledge and a willingness to help.”

“…to not be afraid to approach any given situation and not be frightened by the idea of mental health issues.”

This was indicated to be of particular importance due to shared concern amongst participants about the mental health of multiple groups who engage in community rugby:

“young males are an at risk group and I feel more confident to assist them if needed.”

“I think this will help in my club, which is predominantly teenage girls in minority groups, in identifying girls who are struggling and help intervene early and help them get professional help.”

### 3.5. Delivery Mode 

One area of improvement suggested by participants was the allocation of more time to simulating the skills participants had learnt in the MHFA course:

“I would have liked more time working through scenarios putting the work into practice.”

However, in line with the quantitative findings, the online learning approach was well received due to a perceived increased accessibility and the ability of participants to work at their own pace and in the comfort of their own home:

“really good via zoom (and our only option at present).”

“I was surprised how effective the zoom meetings could be with the breakout rooms etc.”

“it was convenient and practical to go through mental health issues in the safety of your own home was comforting.”; “very good to be able to learn something whilst still in your home environment.”

“I liked this as I could do the e-learning at my own pace and the face to face was broken up over a few days so wasn’t a huge info dump.”

## 4. Discussion

The primary aim of this study was to investigate the effectiveness of online MHFA training in enhancing participants’ knowledge and understanding around mental health, increasing their perceived ability to manage mental health crises, refer individuals suffering from mental illness to appropriate support systems, and reducing stigma around mental health in the rugby community. Following the MHFA training, the largest improvements were seen in the participants’ understanding of knowing what advice to give and being able to identify signs and symptoms of someone struggling with their mental health. Early identification is demonstrated to be an important factor in effective management of mental ill-health [31]. Thus, having systems encouraging practitioners to be well informed on early indicators of mental illness may aid early identification and lead to prevention of severe mental illness in young individuals.

A major barrier to early help seeking in individuals suffering from mental illness is stigma [32]. Participants in this study highlighted that the MHFA training provided an opportunity to challenge the existing stigma, in and around the club rugby community. Enhancing mental health literacy has previously been demonstrated to reduce stigma through the normalisation of discussion about mental health and support seeking in the sporting environment [33]. The present findings indicate mental health literacy of key staff may be essential to creating a community in which the potential barrier of stigma is challenged and minimised. Furthermore, belief in the effectiveness of therapy of both the influential staff and the individual experiencing mental health challenges is a key factor in engagement with, and effectiveness of, support services. Participants reported the MHFA training increased their belief in the effectiveness of therapy provided by a healthcare professional for individuals with mental health problems. 

Participants also reported feeling better prepared to help members of their own community by knowing what to say to someone experiencing a mental health crisis. Such changes are likely associated with individuals’ enhanced ability to recognise mental illness after having completed MHFA training [11,13]. It is acknowledged this study was conducted considering one sporting code (rugby union) with a small sample size, and thus further research with larger samples, and across broader sporting populations, is required to determine the efficacy of MHFA across other sports. Furthermore, as the research was conducted in Australia, the potential influence of location, societal, and cultural influences on the efficacy of the program should considered. Given a number of mental health determinants can be influenced by such factors, this should be considered in both practice and evaluation in future research [34]. However, despite these potential limitations, the present findings are consistent with the changes reported by Australian Rules Football coaches [14] and communities working with young people [17] following MHFA training. Research suggests young people are more likely to seek help in relation to mental illness when they have pre-established and trusted relationships with potential help providers [9]. Thus, proper positioning of staff with the appropriate knowledge and communication skills may facilitate early intervention given the existing relationships between staff, volunteers, and young members. Beyond the field of sport, prior findings consider the importance of improving the confidence of relevant individuals in supporting youth with mental health challenges [35]. Further research is required within the sporting environment to identify the potential sequalae of increased practitioner confidence in aiding the creation of mentally healthy environments in sport. 

The second aim of this study was to evaluate the feasibility of the online delivery mode as a method for MHFA training in community sporting organisations as well as users’ engagement with it. The potential of the influence of self-selecting bias, with participants volunteering to partake in the MHFA training and evaluation being more likely to be motivated and actively engaged in the e-learning modules and online learning activities, is acknowledged. Nevertheless, the online modality was well received, due to a perceived increased accessibility to MHFA material. The online delivery mode was deemed to be as effective as face-to-face delivery with similar outcomes found for knowledge and confidence outcomes, as previously reported in a recent meta-analysis on the effectiveness of MHFA training [36]. Furthermore, the present findings indicate the online learning modality may have given participants additional confidence in challenging stigma surrounding mental illness as they were able to have conversations in the safety and privacy of their own home rather than public spaces. Further research on whether additional benefits and engagement are gained when completing mental health education in a place participants feel familiar and comfortable with is required to further investigate this proposition. An area for improvement in the online delivery mode, as suggested by participants, is the provision of more time spent simulating the skills learnt in the MHFA training, in particular, more time working through mental health scenarios that are likely to be experienced in the future. Allocating additional time to simulating relevant scenarios within the curriculum in future online MHFA courses should thus be considered and evaluated. Overall, the findings indicate that the online intervention increased the mental health literacy of the trainees, and consequently, it is a suitable delivery mode and is economically sustainable for the rugby community, which spans a large geographical area. While online delivery has produced short-term benefits, the long-term direct and indirect benefits of this delivery mode require further investigation.

### Proposed Integration of MHFA into Sporting Communities

A recent International Olympic Committee consensus statement on the mental health of elite athletes has made several recommendations regarding mental health training for coaches. These include providing evidence-based mental health coach education training and encouraging sport governing bodies to demonstrate to coaches that mental health is viewed as an organisational priority by rewarding athletes’ wellbeing as well as win–loss record [33]. While these recommendations focus on elite athletes, they apply to all levels of sport, from the community to the elite level. Recommendations from the present findings include the following:

1. Establishment of a culture that endorses mental health and wellbeing for everyone within the sporting organisation community.
2. Facilitation of an environment that positions periodisation of mental health as an integral part of achievement of sporting excellence.

A number of practical approaches exist that may aid in implementing the aforementioned recommendations, including the integration of MHFA education, promoting visibility and accessibility to mental health information, normalisation of conversations around mental health, positioning of mental health as an essential part of performance, and clarification of organisational responsibility for the creation of sustainable mentally healthy environments. Given sports exist over large, and varied, geographical areas, consideration must be given to the effective and sustainable delivery modes of MHFA training. Available methods of MHFA training delivery include face-to-face and blended, which involves a combination of online courses (e-learning) and an instructor-led component, which is delivered face-to-face or entirely online (e-learning), and an online workshop. The present findings provide evidence in support of a suitable method of online delivery of MHFA training that maximises community sport engagement with a broad geographical reach. 

## 5. Conclusions

This study adds to the existing literature demonstrating that MHFA training is associated with improved mental health literacy and a decrease in stigma in the rugby community. Online MHFA training was found to be an effective mode of delivery and maximised geographical reach. In view of these findings, sporting organisations should consider encouraging their employees to engage with MHFA training, especially in the community club environment. 

## Figures and Tables

**Table 1:** Post-MHFA training outcomes presented in order of strongest average agreeance.

Domain	Mean ± SD Likert Score *
Knowledge to refer others to seek support	4.77 ± 0.43
Knowledge of signs and symptoms	4.71 ± 0.46
Knowledge about provision of initial help for those struggling with mental health problems	4.71 ± 0.46
Useful information about depression and anxiety	4.68 ± 0.48
Knowledge about effectiveness of treatment for mental health problems	4.61 ± 0.50
Knowledge for provision of MHFA support in a crisis situation	4.61 ± 0.50
Useful information about substance use	4.58 ± 0.50

* Likert response options: 1 = strongly disagree, 2 = disagree, 3 = unsure, 4 = agree, 5 = strongly agree.

## Data Availability

The data and survey presented in this study are available on request from the corresponding author.
